# *Salmonella* and *Campylobacter* spp. in Northern Elephant Seals, California

**DOI:** 10.3201/eid1112.050752

**Published:** 2005-12

**Authors:** Robyn A. Stoddard, Frances M.D. Gulland, E. Rob Atwill, Judy Lawrence, Spencer Jang, Patricia A. Conrad

**Affiliations:** * University of California, Davis, California, USA; † The Marine Mammal Center, Sausalito, California, USA

**Keywords:** Campylobacter, Enterobacteriaceae, Pinnipedia, Salmonella enterica, zoonosis, dispatch

## Abstract

*Campylobacter* and *Salmonella* spp. prevalence and antimicrobial drug sensitivity were determined in northern elephant seals that had not entered the water and seals that were stranded on the California coast. Stranded seals had a higher prevalence of pathogenic bacteria, possibly from terrestrial sources, which were more likely to be resistant.

A limited number of surveys have shown that pinnipeds (seals, sea lions, and walruses) can be infected with zoonotic enteric bacteria, including *Salmonella* and *Campylobacter* spp. and that some strains are resistant to antimicrobial drugs (*1**–**3*). Because both *Salmonella* and *Campylobacter* spp. are important zoonotic organisms, their presence in marine mammal feces raises concerns regarding risks to human health associated with exposure to coastal waters and marine mammals. Another concern is that these bacteria in marine mammals may reflect pollution of the California coast by feces from terrestrial sources, including sewage and runoff that contain domestic animal waste. To address these concerns, more detailed data on bacterial pathogen distribution along the California coast are needed.

Northern elephant seals (*Mirounga angustirostris*) are born on various California beaches and do not leave the beaches for several months after birth (*4*). Once the seals leave their natal beaches, they are at sea for most of their lives other than during breeding and the annual molt or if they are found "stranded" (if poor health or injury prevents them from leaving the shore) (*5*). We investigated the prevalence and antimicrobial drug sensitivity of *Salmonella* and *Campylobacter* spp. in northern elephant seals at different sites in California to ascertain the distribution of these bacteria in pinnipeds and determine their potential effect on marine mammal and human health.

## The Study

In February and March of 2003 and 2004, 165 northern elephant seals, which had been recently weaned and had never entered the water, were sampled on their natal beaches at 3 colonies in California (Figure). From February to July in 2003 and 2004, 195 juvenile northern elephant seals were found stranded live along the California coast, rescued, and brought to The Marine Mammal Center (TMMC), Sausalito, California, for rehabilitation. At TMMC, seals were physically restrained and examined, and rectal swabs with Cary-Blair transport medium (BD Diagnostics, Franklin Lakes, NJ, USA) were collected for bacterial culture. Animals were not treated until after sampling.

**Figure:**
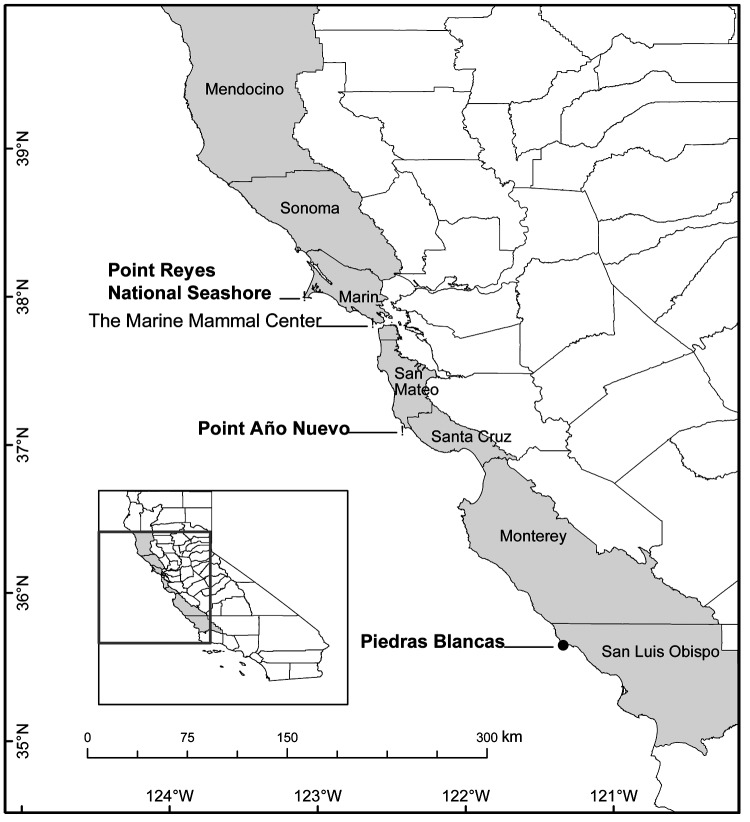
Location of The Marine Mammal Center (TMMC), rescue range of TMMC (shaded), and northern elephant seal rookeries (Point Reyes National Seashore, Point Año Nuevo, Piedras Blancas) where seals were sampled along the California coastline.

*Salmonella enterica* was isolated, identified with standard procedures (*6*), and stored at –80°C in Microbank bead vials (Pro-Lab Diagnostics, Austin, TX, USA) until it was tested for antimicrobial drug susceptibility. *Salmonella* isolates were sent to the National Veterinary Services Laboratory (Ames, IA, USA) for serotyping. Antimicrobial drug susceptibility was performed with broth microdilution according to the Sensititre user manual and National Committee for Clinical Laboratory Standards (NCCLS) guidelines (*7*) for amikacin, amoxicillin-clavulanic acid, ampicillin, cefazolin, ceftiofur, ceftizoxime, chloramphenicol, enrofloxacin, gentamicin, tetracycline, ticarcillin-clavulanic acid, and trimethoprim-sulfamethoxazole. *Campylobacter* spp. were selected by using Campy-CVA agar (Hardy Diagnostics, Santa Maria, CA, USA) that was incubated under microaerophilic conditions at 37°C for 48 to 96 h, identified by using standard procedures (*8*), and stored at –80°C in Microbank bead vials until they were tested for antimicrobial drug susceptibility. *Campylobacter* isolates were tested for susceptibility to ciprofloxacin, doxycycline, erythromycin, and gentamicin according to previously described techniques (*9**,**10*). Odds ratios were compared among the different bacteria and seal populations by using mixed- or fixed-effects logistic regression, with geographic location as the random effect and seal rookery as the fixed effect (*11*). A forward-stepping algorithm was used, and terms with p<0.05, based on the Wald statistic, were included in the final model.

*Salmonella* spp. were isolated from 3 (1.8%) of 165 natal-site elephant seals and 72 (36.9%) of 195 stranded seals. Stranded seals were 41× more likely to test positive for *Salmonella* spp. than natal-site seals (odds ratio [OR] 40.9, p<0.001, 95% confidence interval [CI] 7.7–218). All 3 *Salmonella* isolates from the natal-site seals were serotype Typhimurium and were sensitive to all antimicrobial drugs tested (Table 1). Eighty-three *Salmonella* isolates of 5 different serotypes, Newport, Saint Paul, Montevideo, Typhimurium, and Reading, were collected from 72 stranded seals; Newport was the most common serotype. Eleven stranded seals were positive for 2 different serotypes. Only 4 *Salmonella* Newport isolates from stranded seals were resistant to antimicrobial drugs; 3 isolates were resistant to ampicillin, with intermediate resistance to ticarcillin-clavulanic acid, and 1 isolate was resistant to amoxicillin–clavulanic acid and cefazolin.

**Table 1:** Serotypes and antimicrobial drug resistance* of Salmonella spp. isolated from natal-beach and stranded northern elephant seals, California, 2003–2004

Serotype	No. resistant isolates/total (%)	
	Natal-beach seals	Stranded seals
Newport	0/0	4/42 (9.5)
Saint Paul	0/0	0/17
Montevideo	0/0	0/15
Typhimurium	0/3	0/2
Reading	0/0	0/7
All serotypes	0/3	4/83 (4.8)

*Intermediately resistant or resistant to >1 antimicrobial drugs.

On the basis of biochemical analysis, *Campylobacter jejuni*, *C. lari*, and an unknown *Campylobacter* sp. were isolated from both groups of elephant seals (Table 2). *C. jejuni* was the most common *Campylobacter* species isolated, followed by *C. lari* and the unknown *Campylobacter* sp. (Table 2). One natal-site seal and 8 stranded seals were infected with 2 *Campylobacter* spp. Stranded seals were 6.0× more likely to test positive for *Campylobacter* spp. than natal-site seals (OR 5.97, p<0.001, 95% CI 4.2–8.4). Stranded seals were 4.3× more likely to be positive for *C. jejuni* (OR 4.33, p<0.001, 95% CI 1.8–10.6), 7.2× more likely to be positive for *C. lari* (OR 7.2, p<0.001, 95% CI 2.4–21.4), and 22× more likely to be positive for the unknown *Campylobacter* sp. (OR 21.9, p = 0.003, 95% CI 2.9–164) than natal-site seals. Ciprofloxacin was the only antimicrobial drug to which isolates were resistant (intermediate or complete); resistance was detected in both groups of seals but was more common in stranded seals (Table 2).

**Table 2:** Prevalence and antimicrobial resistance* of Campylobacter spp. isolated from natal-beach and stranded northern elephant seals, California, 2003–2004

Species	Natal-beach		Stranded†	
	Positive seals/total (%)‡	Resistant isolates/total (%)	Positive seals/total (%)‡	Resistant isolates/total (%)
*Campylobacter jejuni*	17/165 (10.3)	0/16§	54/194 (27.8)	2/54 (3.7)
*C. lari*	5/165 (3.0)	2/5 (40.0)	26/194 (13.4)	8/23§ (34.8)
Unknown *Campylobacter* sp.	1/165 (0.6)	0/1	23/194 (11.9)	12/20§ (60.0)
All *Campylobacter* spp.	22/165 (13.3)		94/194 (48.5)	

*Intermediately resistant or resistant to >1 antimicrobial drugs.

†The presence of *Campylobacter* spp. was not determined for 1 stranded seal because other bacteria overgrew on the plate.

‡Number of seals positive can be fewer than total number of *Campylobacter* isolates because multiple *Campylobacter* spp. were isolated from some seals.

§Not all isolates were tested for susceptibility because of contamination or lack of growth after freezing.

## Conclusions

Prevalence of *Salmonella* and *Campylobacter* spp. was higher in juvenile northern elephant seals that became stranded along the coast of central California than in seals on their natal beaches that had never entered the water. A potential explanation for this difference is that stranded seals may have harbored bacteria but were not shedding them while they were in good health on their natal beaches. Infections with some pathogenic bacteria may be asymptomatic, but animals may intermittently shed bacteria, especially if stressed (*12**,**13*). Stress and malnutrition can suppress immunity, which makes an individual animal more susceptible to infection and prolongs existing infection (*14*). Another possible explanation for the higher prevalence in stranded seals is that stranded animals are more susceptible to infection, because of stress or malnutrition, by pathogens in the environment from terrestrial sources, such as contaminated freshwater and sewage outfall. The fact that isolates from stranded seals tend to be resistant supports this possibility.

The cause of the higher prevalence of pathogenic bacteria in stranded juvenile northern elephant seals should be determined, especially if animals are infected by pathogenic bacteria from terrestrial sources contaminating the marine environment. Coastal freshwater runoff is associated with a high risk for infection of southern sea otters (*Enhydra lutris nereis*) with *Toxoplasma gondii* in California and might also be a risk factor for infection of elephant seals with pathogenic fecal bacteria (*15*). Further studies to identify environmental risk factors for infection of elephant seals with *Campylobacter* and *Salmonella* spp. and genetic fingerprinting of these isolates may help determine the sources of these bacteria.
